# Royal Albert Asylum at Lancaster

**Published:** 1907-01-26

**Authors:** 


### ROYAL ALBERT ASYLUM AT LANCASTER.

This is a training school for feeble-minded children; and it admits free patients, between the ages of six and fifteen, whose friends are proved to be unable to meet the lowest payments, and who belong to the counties of Lancashire, Yorkshire, Cheshire, Westmorland, Cumberland, and Northumberland. These children are elected by the subscribers for a stated number of years, while paying cases are admitted without election at charges varying according to attendant circumstances.

Last autumn the asylum issued its forty-second annual report, from which we learn that during the thirty-six years ending in 1906, it had admitted 2,663 children; that 396 of these were discharged much improved; 532 moderately improved; and 363 slightly improved. At the present time the institution has accommodation for 700 children, and there are 650 in residence. Of these, 338 are returned as attending the schools in which an attempt is made to teach, and, as a matter of fact, many do actually learn, the three "R's"; and they obtain some knowledge of the value of money, and are further encouraged by lessons in musical drill. The school buildings are new, and the institution is indebted for them to the kindness of Mr. Herbert Storey, an example which ought to stimulate other residents in the Northern counties, more especially at the present moment when more subscribers are wanted, and more donations needed to complete the memorial building now in process of construction.

During the year 1906 a great loss was sustained by the death of Mr. James Diggens. He had been Secretary since 1865, and on the resignation of Dr. Shuttleworth in 1893 he was appointed "Principal." The Committee bestow well-merited praise on his energy, and they attribute much of the success of the asylum to his efforts and conspicuous ability. All of this is no doubt true; but it is equally true that the Royal Albert Asylum received its great impetus on the way to fame from the first medical superintendent, Dr. George Shuttleworth.

We are glad to find that the Committee have reverted to the only entirely satisfactory system of management, and have again placed the asylum under Dr. Archibald Douglas, as medical superintendent, and it is a tolerably safe prediction that the greater the power with which that officer is armed, the greater will be the ultimate success of the institution.

The committee are perpetuating the remembrance of their late secretary by the erection of a reception-house, which will bear Mr. Diggens' name. In this house all newly-admitted children will be kept in quarantine for fourteen days. We look upon this as a most useful adjunct to such an institution, and we hope that the amount required to finish the work will be speedily forthcoming.

